# Clinical and radiological presentation of a child with progressive pseudorheumatoid dysplasia

**DOI:** 10.1186/1546-0096-12-S1-P167

**Published:** 2014-09-17

**Authors:** E Atsali, KD Stathopoulos, I Bournazos, E Damianou, AA Partsinevelos, Y Dionyssiotis, A Papadopoulou, V Papaevangelou, PJ Papagelopoulos, G Skarantavos

**Affiliations:** 1 3rd Pediatric University Clinic, “Attikon” University Hospital, Haidari, Athens, Greece; 2 Bone Metabolic Unit, “Attikon” University Hospital, Haidari, Athens, Greece

## Introduction

The role of WNT pathway in bone formation and maintenance has been extensively studied since the identification of mutations in key signalling WNT mediators in diseases with high or low bone-mass phenotypes. In humans, loss-of-function mutations in WISP3, encoding Wnt1-inducible signaling protein 3, cause progressive pseudorheumatoid dysplasia (PPD), an autosomal recessive form of spondyloepiphyseal dysplasia tarda.

Individuals with PPD appear normal at birth, have subtle clinical symptoms by 3 years of age and develop later a severe degenerative joint disease with multiple contractures and reduction in joint mobility. Radiologically patients with PPD have multiple sites of epiphyseal enlargement in addition to platyspondyly.

## Objectives

We present a teenage girl with PPD diagnosed as suffering from Juvenile Idiopathic Arthritis (JIA).

## Methods

A 13.5-year-old girl, diagnosed as suffering from polyarticular JIA when she was 4 years old, presented to our department due to severe pain in both hips and difficulty of walking. She was under treatment with corticosteroids (5 mg prednisolone/day), methotrexate (10mg/week), folic acid (5mg/week) and etanercept (25 mg/week sc). Upon examination the girl was unable to walk on her own. She had severe restriction of mobility of ervical spine, kyphoscoliosis, swelling and contractures of elbows, swelling of metacarpophalangeal and interphalangeal joints of both hands with limitation of extension of the fingers and severe restriction of mobility of coxofemoral joints, wrists and ankles. No active arthritis was detected. The girl had normal facial appearance and intelligence.

## Results

Erythrocyte sedimentation rate, C reactive protein, rheumatoid factors and antinuclear antibodies were normal. Xrays of the hands showed widening of the metacarpal and phalangeal epiphyses and loss of joint space. X-rays of the hips showed degenerative changes with almost complete destruction of the joints. Radiographs of the spine revealed flattening of thoracic and lumbar vertebrae (platyspondylia). No soft tissue swelling or erosions could be seen. Peripheral computed tomography revealed extremely reduced trabecular volumetric density, cortical volumetric density and derived bone strength.

The clinical and radiological findings suggested that our patient was suffering from PPD, a disease often misdiagnosed as Juvenile Arthritis. Genetic testing is currently under way.

## Conclusion

PPD is a non inflammatory skeletal disorder clinically simulating early stages of JIA. Non-inflammatory joint involvement with characteristic radiological findings (ie enlarged epiphyses,and platyspondylia) should raise suspicion for this diagnosis. Early recognition of PPD could spare children of unnecessary treatment and lead to early initiation of appropriate rehabilitaition therapy.

## Disclosure of interest

None declared.

